# I am not my self: Reconceiving identity in rehabilitation care

**DOI:** 10.1177/02692155251415256

**Published:** 2026-01-30

**Authors:** Lily Gryspeerdt

**Affiliations:** 1 5292 University of Manchester, Manchester, UK

**Keywords:** Disability, health psychology, perception, rehabilitation, self-esteem

## Abstract

**Problem:**

How does someone altered by illness adjust their view of themselves and how could rehabilitation help?

**Background:**

Rehabilitation is expanding its scope to become more holistic, beyond a preoccupation with physical functioning, which requires an understanding of the concept of personal identity. One currently employed approach defines the self as expressed through the physical.

**Philosophy:**

Such a view risks reifying the self into something owned. Instead, the change a person experiences with illness or injury is not a shift within themselves but reflects alterations in their interactions with the world. A person is not an internal self, mediated by the form of a body. Instead, a person is a body existing in and experiencing the world, particularly through interactions with others.

**Application:**

This revised understanding is significant in rehabilitation because it increases conceptual clarity, removing the perceived challenge associated with defining the self or personal identity. Moreover, by moving towards an integrated view of self, our perspective shifts, such that when a person says, ‘I have changed’, what we can appreciate is ‘things have changed’, thus reducing the blame on them. Consequently, there is increased hopefulness and better acknowledgement of patients’ social situations.

**Implications:**

The purpose of this is not to police colloquial language but to heed against over-interpreting certain common expressions in ways that lead to increased alienation.

**Conclusion:**

Rehabilitation should understand that no self is lost or transformed, but that there is a social identity which changes with altered circumstances and challenges around a person, who remains themselves.

## Preface

O wad some Power the giftie gie us

To see oursels as ithers see us!

- *To A Louse by Robert Burns*

Robert Burns' poem, though concerning the embarrassing affair of a woman with a louse on her bonnet who thinks the stares and smiles of others are in admiration rather than mirth at her misfortune, cuts to the heart of a central aspect of being human: ‘who am I, as you think of and perceive me?’. If we change owing to illness or altered circumstance, such a proposition gains greater significance, such that we wonder, ‘who am I *now*, as perceived by others?’.

## Background: Identity in rehabilitation

When an illness changes how a person feels, or what they can achieve independently, or alters another aspect of their bodily function, then how should we interpret these shifts in the context of their rehabilitation? The changes they experience when unwell can lead to feelings that their body has become a foreign country, their mind an island, while clinicians and family members seek to try to understand them.

The current position within the rehabilitation^1,2^ literature is that the issue of identity is important, but the exact nature of identity being considered is not always clear.^3–5^ Where it is expressed more explicitly, the view of identity used derives from phenomenology^6^ and the philosophy of Maurice Merleau-Ponty,^7,8^ and is opposed to the dualism of Descartes.^9^

It has long been recognised that to have a truly holistic approach, the self must be considered.^10^ This is stressed in the research around neuropsychological rehabilitation, particularly around brain injury,^11–13^ which is the most common area of rehabilitation literature to be concerned with the self. In focusing on identity as key in both injury and recovery, we shift our focus from a purely biological view to consider the social, psychological and spiritual as well. Moreover, identity can help unite these aspects, recognising the way biological damage along with psychosocial factors contribute to a patient's condition.^14^

Talking about identity allows researchers and clinicians to acknowledge the wider impact of illness on patients, such as their biographical disruption, change of sense of self and altered feelings of security.^15^ Such a perspective allows clinicians to tailor their practice or, as Ellis-Hill puts it: ‘people do not always want us to “solve a problem”; being heard, sharing experiences and receiving validation is just as important’.^16^ One systematic review found there is some positive evidence for the effectiveness of using identity reconstruction as a central part of neuropsychological rehabilitation,^17^ although studies are limited.

**Figure 1.**
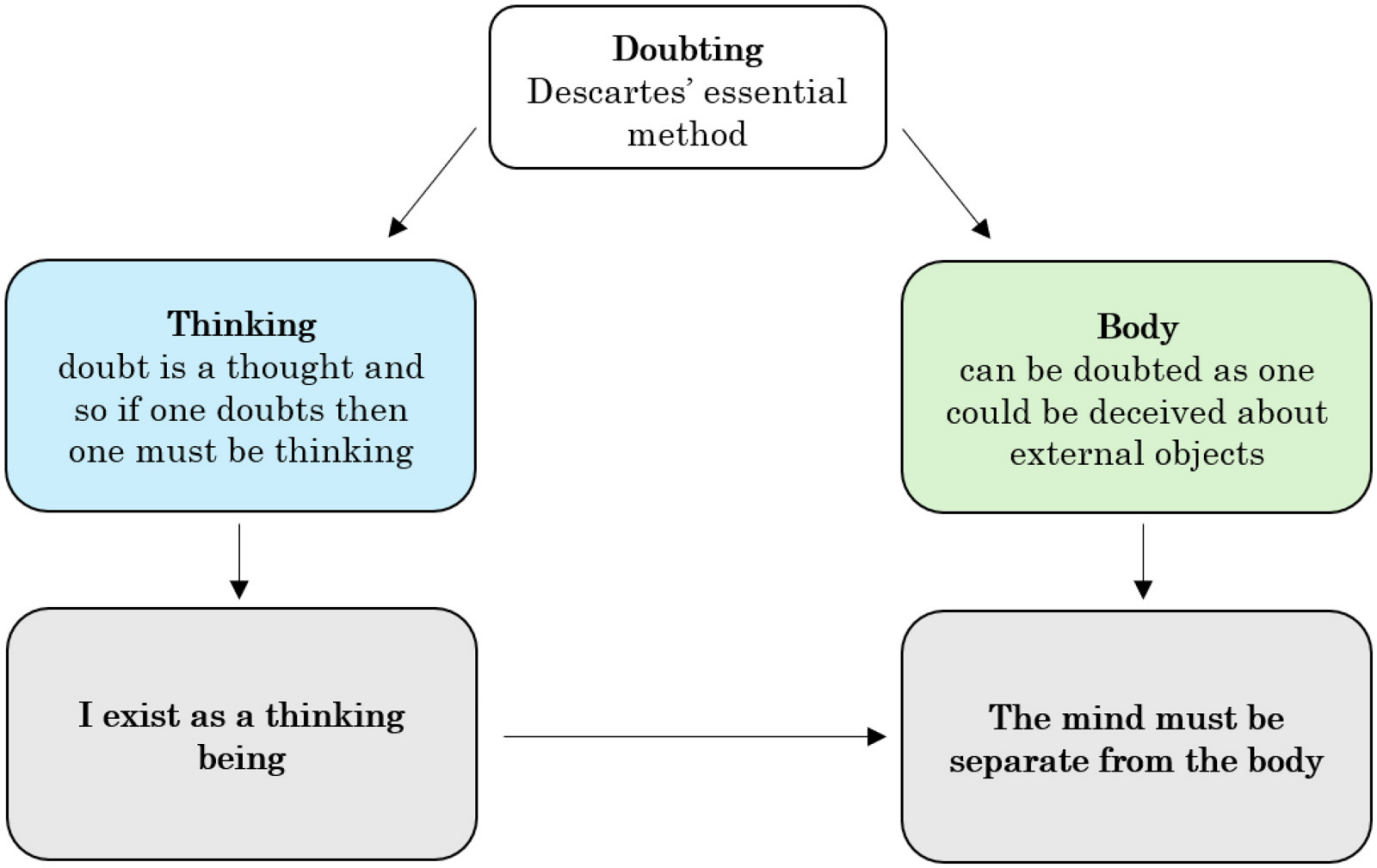
A schematic diagram of Descartes’ process of argument for mind-body dualism. Descartes’ argument begins from the position of doubting. However, in doubting he knows he must be thinking; this one thing is indubitable. Conversely, the body and external objects can be doubted. This leads to the assertion that a person is essentially a thinking being, and that this thought must be separate from the body.

One issue in the literature centres on the problem of incorporating identity into clinical rehabilitation practice owing to the difficulty of conceptualisation.^5^ Definitions of the self in Western philosophy often begin with Descartes’ immortal utterance of the cogito: ‘I am thinking therefore I exist’.^9^^, p. 36^ This is derived from Descartes’ doubts in his senses such that they should not be trusted as leading to knowledge. However, in declaring this he observes ‘it was necessary that I, who was thinking this, was something’,^9^^, p. 36^ thus leading to his assertion that the self is distinct from the body and resides in the intellect, forming the sole thing one cannot coherently doubt. The immaterial self which gives rise to thinking is the source of being and certainty to Descartes^18^ (see Figure 1 for a representation of this argument).

On the surface of it, the Cartesian dualist view fits with the experience of brain injury. In these cases, the seat of change is the mind. If a person's mind is altered then so is their self, thereby inferring that the self and the mind are one and the same. However, this mind-alteration is the result of a physical process, such as trauma or a neurological change. This connection between the physical and the mental has been of difficulty for dualists since Descartes first proposed the theory.^9^^, pp. 230–238^ The relationship between the brain, as a physical entity, and the self that is altered in injury is of challenge to the dualist view as it suggests the body is the reason for the mind's existence.

Moreover, other areas of rehabilitation are involved in identity change, without being linked to the brain or mind. One of the first ways I was confronted with the struggles of identity in rehabilitation as a medical student was in speaking to a woman, Maria (not her real name – see Supplementary Data, Case One) who had had a mid-knee left leg amputation five months previously and was really struggling to come to terms with it. She was still in pain, fearful of losing her other leg and frequently tearful. She was worried about caring for her son and what it would mean for her work. With the loss of her leg, she felt like so much had been taken, including many activities from which she gained a sense of identity. Her mental processes had not been interfered with by her condition, however, the physical reality she was coming to terms with had altered her selfhood.

Despite this rejection of dualism, the notion that the self is important has persisted. Although there is a reluctance to pin down a specific view of the self in rehabilitation, with many taking a ‘hyphenated’ view which encompasses many schemas,^3^ a phenomenological approach has proved popular.^19–22^ Derived primarily from Merleau-Ponty,^8^ this view takes experience as subjective perception, with a quality known only to the proprietor, but which is inherently situated within a body.

As per phenomenology, the focus of understanding is through consciousness.^6^ This has a necessarily subjective quality as it is inextricable from a person's individual experience. However, unlike Descartes’ dualism, this experience is not separate from the body but only possible because of the body. Therefore, a human has a subjective self in the form of a body, and it is through this that one exists in the world.

Consequently, having a body is a necessary condition of personhood, such that it is through the body one can exist in and with the world. In contrast to Descartes, this view stresses the realness of the physical and its unity with the intellectual: ‘The world is not what I think, but what I live through. I am open to the world, I have no doubt that I am in communication with it, but I do not possess it; it is inexhaustible’.^8^^, pp. xviii–xix^ This means the body is both an object in the world and a subject through which the world is experienced.^22^

**Figure 2.**
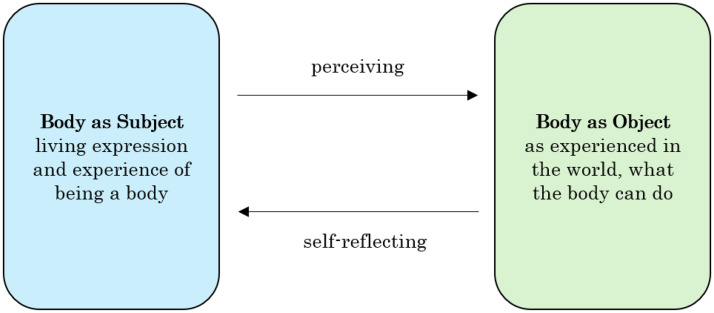
A schematic diagram representing Merleau-Ponty's view of the self. According to Maurice Merleau-Ponty, there are two aspects to the self: the body as an object in the world and the body as the subject through which the world is experienced. The body as object relates to the subject through self-reflection; the subject relates to the object through perception. These two aspects are not separate, as in dualism, but are unified, like two sides of the same coin. Developed with reference to.^23^

As a result of this, the body is the essential means by which the world is experienced but we also experience the body in the world. For example, when one hand touches the other, I am experiencing my body within space and time.^7^ Similarly, when I see, it is from the perspective of my eyes. See Figure 2 for an illustration of this relationship, which composes the unity of the body and the mind. Note that the body as subject and body as object are different ways of experiencing the same thing, not as dualistic substances.

In discussions about Merleau-Ponty's view of the body and its relation to selfhood, this is often focused on ‘I can’^7,24^ as the source of identity. As the experience of perception involves the body, and our implicit self-awareness of it, we define ourselves in terms of our bodily abilities. By this it means that our conscious self is not based in the mental but in a harmony between what we can do and the world we do it in.^24^ Thoughts about ‘self’ then proceed from this. For example, when the woman with the amputation, Maria (Supplementary Data, Case One), described above, wakes up and gets out of bed, her experience in the world is defined by the absence of that leg and so this bodily change defines a change in her selfhood. The vessel through which she exists in the world is altered (the object) and so too is her internal experience of the world, which defines her perception and identity (the subject).

## Method: A philosophical alternative

These philosophical underpinnings, though, are not incontrovertible. While phenomenology has proved beneficial in taking seriously the role of the body in identity, there are some short-comings in this method, such as its continued separation of the person from the social setting. Consequently, I will propose a different philosophical method, based on the later work of Ludwig Wittgenstein,^25–27^ which will then serve as a basis for re-situating discussions of identity within the social settings that patients find themselves in.

My critique of the established view stems from two motivations: one philosophical, and one based in experiences of seeing people engage with rehabilitation care. In this section, I will expose this philosophical underpinning in particular, but with the experiential motivation and influence woven throughout.

Under Merleau-Ponty's view the body is the thing through which the self becomes known, and through which the self is, in part, constructed. While this is appealing, why must we consider there to be an ethereal ‘self’ to be known or constructed at all? The phenomenological view is received positively for giving a more unified understanding of body and self, however, in still acknowledging some kind of separation it is in danger of continuing the dualism of the Cartesian cogito. In thinking that I am my self, as known and able to know through my body, there is still some separation in how we view the inner and outer lives that a person lives. A distinction is still being drawn which risks leading us into confusion, akin to that of Descartes’ cogito, and away from the conceptual clarity required to integrate understandings of identity into rehabilitation practice.

The origin of this mistake can be seen in certain ways we are naturally tempted to think but which lead us to incorrect conclusions. Our appreciation of someone's personhood rests in our acknowledgement of their self; through appreciating that they have an individual experience of the world. This can also be seen in the terms of our own inner experience, without which the me that I am feels like it would not exist. In having this sense of self there is a temptation to begin to believe that this self is, in some way, separable from other facets of my person. We are drawn into thinking that, because we think there is no person without a self, the self must be identifiable as separate from the person.

This confusion is one which Wittgenstein identifies in his *Philosophical Investigations*. For Wittgenstein, the construction of the self arises from a mistaken philosophical analysis^26^ (§413) in which we are misled by the grammar of phrases like ‘I am in pain’ to believe this signifies an inner self by whom the pain is experienced^26^ (§404–410). We take the ‘I’ not as a replacement for my name or my whole person but a self who experiences that pain and about whom we truly refer when we talk of a person's identity.^27^ In Descartes’ terms this is the soul, while to Merleau-Ponty this is the subjective perception of a body. Accordingly, there is not an ‘I’ who is perceiving the world through having a body; it is just that I perceive the world as a body. This is not some form of hidden inner reality separate from outer appearances but the exact same thing; one person.

**Figure 3.**
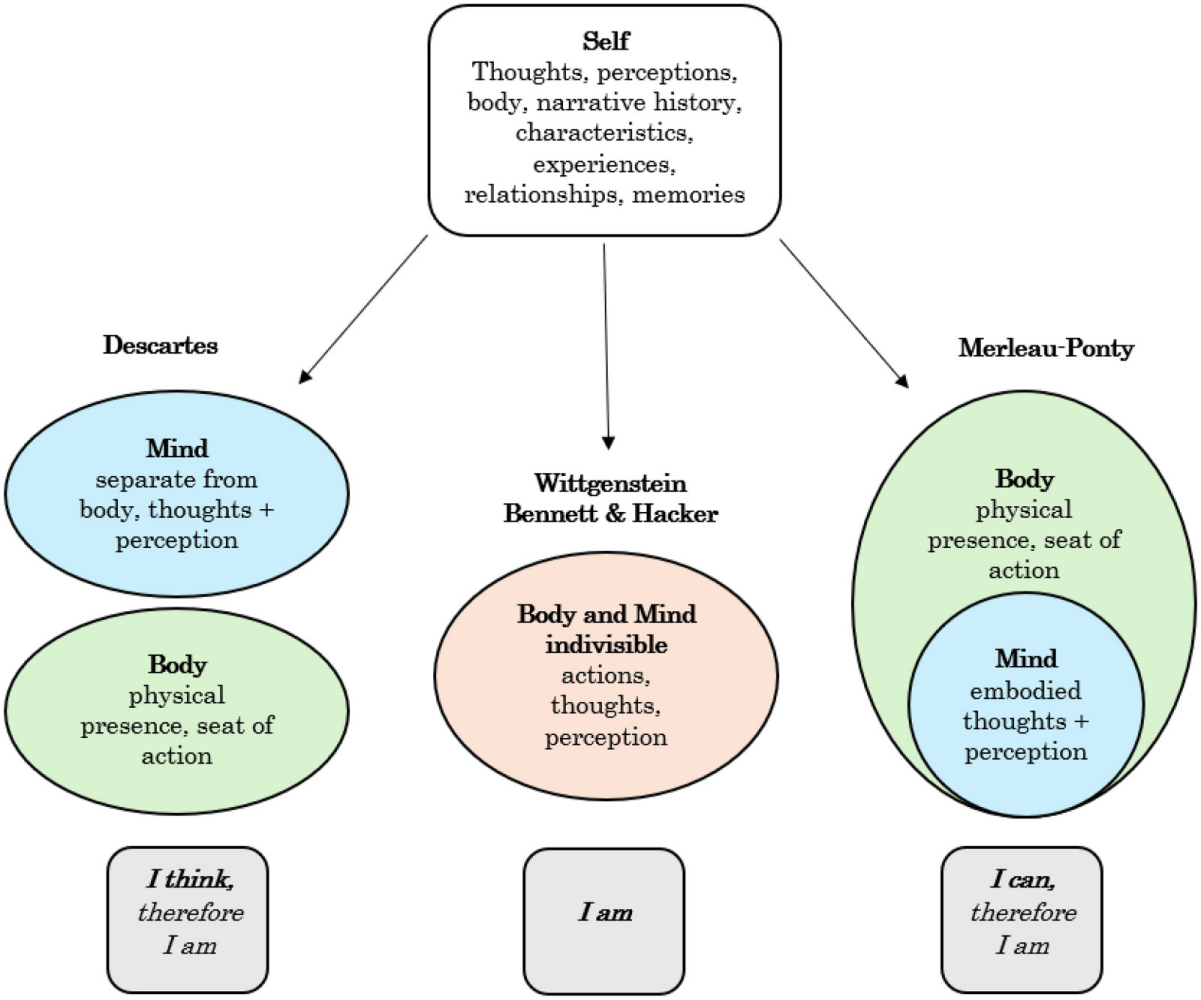
Three views of the relationship between the self, the body and the mind three different understandings of the self. Firstly, Cartesian dualism, where the mind and the body are separable and distinct, with the mind as immaterial. Contrasted with this is Merleau-Ponty's phenomenological perspective which defines the self in terms of an embodied mind. Finally, the view of Wittgenstein and Bennett & Hacker who reject reducing the self or creating divides in our definitions.

This view can be seen elucidated in the schematic, Figure 3, which shows the Cartesian, Wittgensteinian and phenomenological explanations of the self.

This is further elicited in Bennett and Hacker's *Philosophical Foundations of Neuroscience* which describes the notion of the self as ‘an aberration’ resulting from the confusion of tacitly inserting a space into reflexive pronouns such as ‘myself’, ‘yourself’, ‘ourselves’ to make ‘my self’, ‘your self’ and ‘our selves’. Consequently, ‘having opened up an illicit space, we then fall into it’.^28^^, p. 382^ Such a misunderstanding entices us to believe that there is a ‘mysterious object’ called a self ‘whose nature must be investigated’.^28^^, p. 382^ We create a psychological Ego, to be defined, rather than accept that ‘to speak of myself is not to speak of a self which I have, but simply to speak of the human being that I am’.^28^^, p. 382^ When thinking about a person there is no separate ‘self’ to be considered: they are them and they are themselves, accepting what we see before us as the entire person.

To think about it a different way: my experience of the world is not conveyed through my body, it is being that body. I exist in the world not as a self, mediated by the form of a body, but instead it is in virtue of being a body that I exist. Moreover, it is important to note that this is a socially-situated body, with relationships to others and existing within a cultural context. The physicist Carlo Rovelli, writing about quantum theory and the nature of reality, says, ‘Individual objects are the way in which they interact. If there was an object that had no interactions, no effect on anything, emitted no light, attracted nothing and repelled nothing, was not touched and had no smell …. It would be as good as non-existent’.^29^^, p. 68^ As a result of this, reality is defined by the interactions of objects, woven together in relationship with one another.

This might appear similar to the Merleau-Pontian phenomenological view owing to its stress on unity between body and self. However, in speaking of the ways in which the body can be understood, and the positioning of the body-as-subject being experienced differently to the body-as-object, Merleau-Ponty is still falling into the trap of creating a self, separate from the experiential. Instead, as Prinz says in his examination of Wittgenstein's philosophy in relation to neuroscience, when introspecting or examining another, ‘We can find thoughts, intentions, and the body in action, but not the bearer of thoughts, the author of intentions, or the owner of the body’.^30^^, p. 158^ Merleau-Ponty still draws a distinction between an inner and an outer life, although he does not go so far as to separate the former into a reified ‘soul’. While he is right to stress the primacy of the body in the existence of a person, he still falls foul of the temptation to still try to explain how there is ‘a self’ at all.

Accordingly, when considering the aforementioned difficulty in conceptualising self-identity for discussions in rehabilitation medicine, the search for meaning is turned on its head. The self is never found because it was never there to be found. We must therefore limit ourselves in what we seek to study, to consider expressions of self as about what is happening in one's life and one's attitude towards life.^31^ In our rush define the ‘self’ we should step back and question whether we are wrong to try and define a *thing* at all.

In short, I am not my self: I am.

## Results: Application to rehabilitation

**Figure 4.**
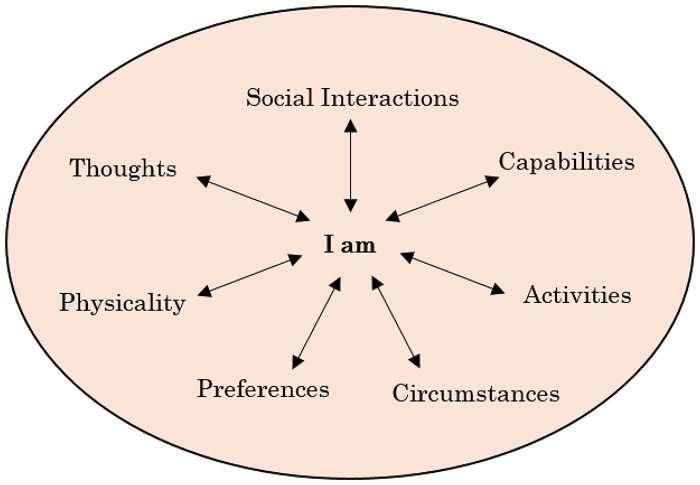
An integrated view of self the aspects here are not intended to be an exhaustive list but a snapshot of the irreducible and interacting elements of a person's existence, such as their physicality (appearance, sex characteristics, gender presentation), thoughts (narrative, concepts), social interactions (relationships with family, friends, acquaintances), capabilities (what they are able to do), activities (what they do), circumstances (situations a person finds themselves in) and preferences (personal tastes, proclivities). Altogether these constitute constitutes the living of a life – the ‘I am’.

The result of this is an integrated view of identity, as illustrated by Figure 4, where the ‘I am’ of our identity (see Figure 3) is lived and interrelated with various different aspects of our lives, such as our social interactions, our activities and our physical body and appearance. This relationship is one which continually grows and develops. There is, therefore, never a static self by which our identity is maintained internally. I am not identified with an internal self but have an evolving relationship with the world around me, shaping and shaped by changes. A life is not lived within a mind but through and with the world around us.

That this concept contains such plurality is intended to show its irreducibility. Its breadth is not indicative of an extended and convoluted definition (such as in the ‘hyphenated’ view proposed by some^3^) but reminds us that there is not a single, separable thing such as the ‘self’ to be defined away from all the rest of the actions, interactions and being of a person. This is a complexity which does not call for us to say more but actually to say less; not to take it apart into its constituents but to remembers its wholeness.

This is not purely a philosophical or theoretical, perspective. It has real application and relevance for rehabilitation clinicians and, more importantly, for patients. To explore this, I will illustrate an experience I had with a woman in her 80s, who I will call Joan, (see Supplementary Data, Case Two) who was coming to terms with a second stroke, and two keys ways the above discussion is important for rehabilitation.

Joan's second stroke had occurred just a couple of weeks before I spoke to her when she was still in the acute stroke unit. She had had her first one four years previously, leaving her left arm paralysed, although she had recovered some of her ability to walk. This second stroke had affected her left side again. Considering her advancing age, she felt pessimistic about her future and whether she would be able to recover some of her strength another time. Since her first stroke, she had been somewhat dependent on the assistance of her son for activities of daily living and she was now looking at a long process of recovery in which she would need to rely on him even more, with her independence further reduced. She felt like so much had been taken, including many activities from which she gained a social identity, like visiting friends.

If asked about how she felt about all this, she doubtless would have said, like many patients do, that she simply did not feel like herself anymore, that she was not the same person as she was before the strokes. Now, from a philosophical standpoint, I might want to challenge this language and dissect what she meant by this, to relocate the source of change she was experiencing.

However, this is not about policing how patients express themselves. The colloquial expressions around the self can still carry meaning for an individual and can be helpful as metaphors for conveying a person's experience. For example, one interviewee in a study talked about their brain injury as the death of their self.^32^ This is certainly expressing something about the reality that the person was living – the profound changes they had undergone, the chasm between their current experiences and their past interactions.

Nevertheless, what is ruled out are philosophical, psychological and neuroscientific claims based on this way of speaking^28^^, p. 384^ which mean that, in talking about the self, the person is taken as making a claim about a thing which exists. It is a patient's experience and emotions which ought to be taken seriously, not any potential metaphysical attribution presumed as a result of this. This is not about correcting how patients express themselves but ensuring their words are not philosophically over-interpreted.

As such, the first way this disavowal of a reified self is of value for rehabilitation medicine is in lending conceptual clarity. This is something that is frequently cited as an important area of development, with conceptual clarity needed to precede further advances in approaching identity in rehabilitation.^5,33^ Some researchers try to get around the complexity of defining the self by speaking of ‘sense of self’ (see ref.^17^). ‘Identity’ is another term used. However, without sufficient clarity, these terms become proxies, always edging close to the idea that there is a self to define or to have a sense of, or that an identity is some*thing* we possess.

Secondly, beyond conceptual clarity, this integrated view of identity as resting not in a self but in a person, whole and complete, is profoundly hopeful within rehabilitation, making this also a practical argument. When a person experiences a significant illness or injury, it is not that their previous self is lost, broken, damaged or changed; instead, their circumstances, reactions to circumstances or physicality have changed.

This is not to downplay its significance but to iterate a sense of continuity. If you have an idea of the self as a ‘thing’ you can realistically talk about it being lost or broken, much like a prized possession. The change the person has undergone is then located *within them* and it is framed as a defect, or it places their previous way of being as out of reach.

Consequently, the picture created is incorrect, negative and lacking hope. As Whiffin and Ellis-Hill ask: ‘How many clinicians have said the words ‘they will never be the same’ to a family member by a hospital bedside?’.^34^^, p. 125^ If we take this within the model of self-as-thing, you are telling the family that some fundamental essence of their relative has altered.

Once again, this can be seen through the woman with the second stroke, Joan, discussed above (Supplementary Data, Case Two). As she spoke of the differences between her life before and after her illness, there was such a sense of loss. However, rather than seeing this as some*thing* lost or altered, a self she no longer possesses, an identity changed, we should view this as an altered way of being in the world; a new relationship to it. What Joan needed was not to construct a new model of selfhood but assistance in adjusting to her different embodied relationship with the world.

Now, consider the following sentences:

1. I have changed.
2. Everything has changed.

To centre discussions around the former is to place the locus of change as entirely within the person, seemingly attributing blame. The latter encompasses the experiences and interaction of others around the person as they can be the cause of change, as much as the illness or disability itself.

Once again, this is not to police the language used colloquially but to allow for critical assessment of the language that is used. To believe you, as a person, are fundamentally changed is profoundly alienating. This is similar to the reasoning of Glintborg, who warns against reifying a diagnosis of brain injury such that it becomes the person's full identity and subjugates their humanity.^33^^, p. 27^

Echoing this, I would warn of the dangers of reifying the self, estranging patients from their continued humanity and focusing discussions on what is lost, not on their persistence. I spoke to a patient I will call Tony, with a recent above-knee amputation (see Supplementary Data, Case Three), and, while he undoubtedly had concerns about the impact it would have on his life, he was still keen to talk about his hopes for the future and the desire to be a good husband, father and grandfather. Owing to simple practicalities, he would not be able to function in the same way as he had previously, even before considering the different judgements and limitations placed on him by others’ perceptions.

To some, this kind of change requires ‘identity reconstruction’. Ownsworth and Haslam, for example, say: ‘Self-continuity is often achieved in the course of re-connecting with one's values, activities (e.g., hobbies) and social networks and roles (e.g., as a parent); many of which are closely tied to one's self-understanding… identity reconstruction ultimately involves coming to terms with an altered lifestyle and change in future goals’.^17^

The way Tony (Supplementary Data, Case Three) was seen in these social roles had changed, and this required some sense-making. Still, I would deny that this is the same as leading to the formation of a new self. Rather, these are changes enough within themselves, without being taken as an indication of an essential alteration. While I acknowledge these changes are significant, I dispute the suggestion that these changes in relations indicate a deeper shift in his personhood, or a precursor to rebuilding his self. Therefore, instead of identity reconstruction, we ought to focus on meaning creation, and finding ways of coping with and understanding the situation the patient is in.

Much of the literature around self-identity in rehabilitation utilises narrative as a key tool.^16,33,35^ It is here I can find some common ground, although rather than seeing narrative as a means for expressing a new ‘inner subjective experience’,^5^ it is constitutive of our senses of personhood, a way of locating ourselves within the social world. Holmes, for example, rejects the idea of a pre-existing self in favour of one which forms through telling: an ‘autobiographical self’.^36^^, p. 179^

In speaking of his hopes for the future, and the things he had done in the past, Tony (Supplementary Data, Case Three) was not just telling me about himself but developing that understanding of who he was and how he relates to the world. This is not just a cerebral activity but one which also relates to the body. His narrative gave me a sense of his bodily capabilities, such as how he used to run marathons, and conveyed his current pain. Through narrative, though, these are not isolated but connected, incorporating how he sees himself as a person and how he believes others see him.

## Discussion: Implications and challenges

The view of identity we have come to, then, is an integrated one: a person is not identified with a reified self but is engaged in a continually developing interaction with the world. There is no singular ‘thing’ which we should define as identity but instead see it as a dynamic process of being in the world. When applied to rehabilitation patients this is beneficial in helping shift the locus of blame away from the individual. Rather than painting a picture of their illness as leading to a loss of some*thing*, we should shift the discourse towards understanding their changed position within and interacting with a social environment.

This view is also in line with the social model of disability, as established by Michael Oliver^37^: the patient is not a defective version of who they once were but they are in a different set of circumstances which pose new challenges, new ways to react and new barriers owing to the way the world is set up and their interaction with it.

Michael Oliver's view was that ‘disability is the disadvantage or restriction of activity caused by a contemporary social organisation which takes no or little account of people who have physical impairments and thus excludes them from participation in the mainstream of social activities’.^38^^, p. 22^ Of course, this is notwithstanding the various critiques levelled at the original social model of disability, like its denial of the body's role,^39^ but to highlight the importance of social relations to a person's experience of their ‘embodied vulnerability’.^40^

This is further supported by a 2014 study which looked at 49 people with mild-to-severe traumatic brain injury and the impact they believed this had on their self-identity.^13^ It found three main themes underpinned this: firstly, having a strong and coherent sense of being a whole person; secondly, respect, validation and acceptance by others; and thirdly, feeling like they have a valued place in the world. Although not using the same language, these themes reflect a similar perspective to that illustrated in Figure 4. Feeling humanised, and having strong relationships, for example, was important to the participants, with social interactions having the power to both build and breakdown this sense of worth.^13^

The view I have presented, though, remains one which can seem counter-intuitive and requires a change in the way we approach conversations about identity. To go back to the argument presented by Bennett and Hacker, it is deeply tempting to take reflexive pronouns such as ‘myself’ as entailing the existence of my *self*.^28^^, p. 382^ This becomes even more troublesome when both affirming the importance of validating the feelings behind a patient saying something along the lines of ‘a part of me died when I had the accident’, and believing that this is not a metaphysically significant statement. These views are appealing and set traps which are all too easy for us to fall into.

As such, the integrated view I have proposed goes against the prevailing narrative around the topic of self and identity. Neurologist Masud Husain's book *Our Brains, Our Selves*^41^ illustrates this dominant stance. Through seven cases based on his patients, he builds an argument wherein the brain is the seat of the self, though not in a particular area but in the community of its parts – or the ‘society of mind’.^41^^, p. 240^ The argument he creates is a simple and attractive one: the brain is the source of our cognitive process, which guide our actions, and thus constitute our selves. We can see this when the brain is damaged in some way, as personal characteristics are subsequently changed, not as a whole but in relation to the specific part which has been subject to the assault.

The problem with this argument is revealed in his explanation of the role of social identity. From the very beginning, Husain acknowledges that as well as ‘personal identity’ we also have ‘social identity’.^41^^, p. 10^ While personal identity consists of our memory, motivation, perceptions and so on, social identity is a matter of how we are perceived by and interact with others.^41^^, p. 2^ To an extent, he argues, the brain is also a source of social identity: I want to fit in and so I learn from others, to develop new cognitive abilities, to allow for thriving or just survival, within social contexts.^41^^, p. 16^

However, if the brain is reacting to others then it becomes a conduit for the influence of others. In this, the brain no longer has the primacy Husain had attributed to it. If social identity is so important for personal identity, and if the two interact, then it is not the case that the self is just the cognitive functions of the brain but, by his own admission, related to something greater.

In focussing on the brain, there is little mention of the role of the rest of the body's role and how our physicality constructs our identity. In his conclusion Husain talks about having a ‘body schema’^41^^, p. 250^ and how this contributes to identity. Given the context, I assume he means by this how we perceive our own bodies. However, what about how those bodies are perceived by others? An important example for this would be race. Someone of a race which is discriminated against in a particular society will have their experiences and identity shaped by that impression, regardless of what their individual perception of their own body. Once again, his neat delineations between brain and personal identity, and social identity begin to blur.

The argument Husain presents is a reductive one – but in this reductionism lies its appeal. We want to be able to tell a singular and simple story of identity, and the view that our brains are our selves is an enticing place to start. He acknowledges, with reference to Baumeister,^42^ that the self is an important cultural concept.^41^^, p. 239^ Yet he then goes on to ask where it is located, how we might boil it down to the brain, and so on. But why might a cultural concept need a location? We ought to no more expect to find a location for ‘self’ than for society, civility, or any other such concept.

As I have argued, identity is certainly a powerful cultural concept, and one which it is important to consider in rehabilitation, but acknowledging it as such does not have to lead to reified or reductive definitions which, subsequently, centre change and defection within those who are ill or injured, without acknowledging the broader context in which they are situated. In this, social relationships are fundamental in developing personhood. Nevertheless, the consequences of this mean that an identity is not something you ‘have’ like a possession, but it is something you live. You *are* your identity; you *are* yourself. In being lived, it is dynamic and can be impacted by various circumstances and changes.

## Conclusion

We are creating a false perception of reality by isolating the ‘self’, even in an embodied sense. Instead, when a person says, ‘I have changed’, what we can appreciate is ‘things have changed’, thus reducing the blame on them. Through a Wittgensteinian rejection of the reified notion of the self, we can understand the wholeness of a person, regardless of their illness or disability. In this, we can also see the importance of narrative: not the reporting of some internal sense of self but the creation of personhood, as an expression of an attitude.

Within rehabilitation, understanding that the self is not separable from the person can help to build a sense of what the patient is experiencing, and what they need. Seeing change not a fundamental shift in who they are in themselves but as an altered place in the world, maintains a sense of continuity. In turn, this aids listening to patients’ needs and ought to encourage hope. It is essential to see this in a social context and as developed through narratives. No self is lost or transformed, but a social identity which changes with altered circumstances and challenges around a person who remains themselves.

**Clinical messages**

- The change a person experiences with illness or injury is not a shift within themselves but in their interactions with the world.
- The idea of a ‘self’ akin to a possession should be rejected in favour of an integrated view of identity as irreducible.
- Social narratives and interactions aid understanding.

## Supplemental Material

sj-docx-1-cre-10.1177_02692155251415256 - Supplemental material for I am not my self: Reconceiving identity in rehabilitation care Supplemental material, sj-docx-1-cre-10.1177_02692155251415256 for I am not my self: Reconceiving identity in rehabilitation care by Lily Gryspeerdt in Clinical Rehabilitation
