# Supplementary material for: I am not my self: Reconceiving identity in rehabilitation care
Source: Clin Rehabil. 2026 Jan 30;40(7):859–70. doi: 10.1177/02692155251415256 (PMC13283497; doi:10.1177/02692155251415256)
Supplement: sj-docx-1-cre-10.1177_02692155251415256 - Supplemental material for I am not my self: Reconceiving identity in rehabilitation care [file sj-docx-1-cre-10.1177_02692155251415256.docx]

**Supplementary Data**

Below are three cases, referred to in the paper, which are derived from real clinical cases, but the details have been altered and names changed for anonymisation. Their purpose is to enhance the understanding given in the main body of text, and to assist with the real-world application of the theories discussed in this paper.

**Case One**

*Maria, aged 43*

*Maria was diagnosed with Systemic Lupus Erythematosus (SLE) more than a decade previously, at the age of 32. Initially this was managed with hydroxychloroquine and occasionally steroids for flares. However, she found her symptoms were no longer adequately responding to DMARDs so trialled on a new biologic therapy.*

*She had a severe reaction to it leading to cellulitis, osteomyelitis, and gangrene in left leg. Despite stopping the drug, her condition progressed, and the decision was made for a mid-knee amputation on her left side.*

*Though this removed the infection, she was left in severe pain and was struggling to mobilise. She had also had some recurrent cellulitis in her right leg, leaving her in fear that this would be amputated too.*

*Aside from rheumatoid arthritis she had type 2 diabetes mellitus and admitted struggling with glycaemic control.*

*She had previously worked as a teaching assistant but had not worked for several months. She was a single mother to a 12-year-old son with autism and struggled at school.*

**Case Two**

*Joan, aged 82*

*Four years ago, Joan had a Total Anterior Circulation Stroke (TACS) which left her hemiplegic on her left side. Since then she had recovered some of her ability to walk although she had continued to have paralysis in her left arm.*

*She then had a second stroke, this time a Partial Anterior Circulation Stroke (PACS) which also affected the left side. She was particularly concerned about her walking and her ability to regain strength in her left side.*

*During her recovery from her previous stroke she had spent several months in a rehabilitation unit. In this time, she had progressed well, although found the process slow and tiring.*

*Since her first stroke she had moved back into her own ground floor flat but with her youngest son nearby to help her with shopping, cooking, and cleaning. She was previously able to complete her own personal care without assistance. Four years ago she had been part of a number of social groups, including knitting and walking, but these had been reduced to once a week coffee mornings owing to her decreased independence.*

**Case Three**

*Tony, aged 75*

*Two weeks ago, Tony had had an above-knee amputation secondary to peripheral arterial disease. He had been struggling with claudication in his legs when exercising for a number of years but this had progressed to pain at rest. His leg had then started showing significant signs of infection and tissue loss. Previous debridement surgery had been partially successful but did not offer a long-term solution.*

*He had been reluctant to have the amputation but considered it the last resort and his best option for being free from recurrent, painful infection.*

*Tony had a 60 pack year history of smoking and even after his amputation he was struggling to quit. He had worked in the hospitality industry for all his life and believed smoking was a big part of his social and professional relationships.*

*He lived with his wife and has two grown up children. He is now a grandfather to five and was keen to be able to be around for them. He retired about a decade ago but still kept in touch with his former colleagues and customers. When he was a young man he had been involved in lots of sports, playing for a local cricket team, and completed a marathon when he was in his 40s. Osteoarthritis had made him less active from when he reached his mid-50s.*
